# AEKF-SLAM: A New Algorithm for Robotic Underwater Navigation

**DOI:** 10.3390/s17051174

**Published:** 2017-05-21

**Authors:** Xin Yuan, José-Fernán Martínez-Ortega, José Antonio Sánchez Fernández, Martina Eckert

**Affiliations:** Centro de Investigación en Tecnologías Software y Sistemas para la Sostenibilidad (CITSEM), Campus Sur, Universidad Politécnica de Madrid (UPM), Madrid 28031, Spain; jf.martinez@upm.es (J.-F.M.-O.); j.sanchez@upm.es (J.A.S.F.); martina.eckert@upm.es (M.E.)

**Keywords:** underwater simultaneous localization and mapping (SLAM), augmented extended Kalman filter (AEKF), FastSLAM 2.0, loop closure, computational complexity

## Abstract

In this work, we focus on key topics related to underwater Simultaneous Localization and Mapping (SLAM) applications. Moreover, a detailed review of major studies in the literature and our proposed solutions for addressing the problem are presented. The main goal of this paper is the enhancement of the accuracy and robustness of the SLAM-based navigation problem for underwater robotics with low computational costs. Therefore, we present a new method called AEKF-SLAM that employs an Augmented Extended Kalman Filter (AEKF)-based SLAM algorithm. The AEKF-based SLAM approach stores the robot poses and map landmarks in a single state vector, while estimating the state parameters via a recursive and iterative estimation-update process. Hereby, the prediction and update state (which exist as well in the conventional EKF) are complemented by a newly proposed augmentation stage. Applied to underwater robot navigation, the AEKF-SLAM has been compared with the classic and popular FastSLAM 2.0 algorithm. Concerning the dense loop mapping and line mapping experiments, it shows much better performances in map management with respect to landmark addition and removal, which avoid the long-term accumulation of errors and clutters in the created map. Additionally, the underwater robot achieves more precise and efficient self-localization and a mapping of the surrounding landmarks with much lower processing times. Altogether, the presented AEKF-SLAM method achieves reliably map revisiting, and consistent map upgrading on loop closure.

## 1. Introduction

Water covers approximately 72% of the Earth’s surface, and oceans and seas are home to almost 90% of all the known species. Therefore, the subsea environment is very interesting for exploration, but also one of the most challenging environments, as robotic navigation is highly difficult due to marine currents, water pressure, low light with different spectrum than daylight, turbidity, etc. Autonomous Underwater Vehicles (AUVs) have been widely used in oceanographic studies and for military purposes for several years. To date, they are increasingly employed to explore rigid and complicated environments like oceans, harbors or at dams, such as using the on-board side scan sonars (SSSs) of the AUVs to image the seabed. Nowadays, the robotic research community has attached great importance to the underwater robotic mapping problem. The most crucial and the most essential requirement for an autonomous robot is having a precise and detailed map. The robot creates a spatial representation of the working environment from a sequence of on-board navigation sensor measurements as it surveys that area. This is generally regarded as one of the most important problems in the pursuit of building truly autonomous robots.

**Figure 1:**
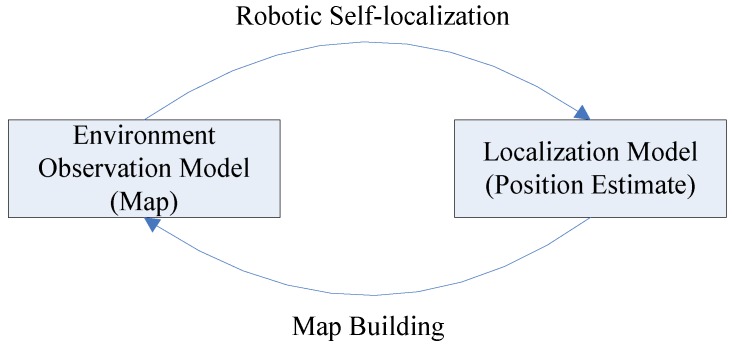
The problem of robotic localization and mapping.

There exists a cyclic nature problem in the robotic mapping and localization: while operating in an unknown environment, in order to build a map of the uncharted territory, the fully autonomous robot needs to know its location, but therefore it requires a map. This chicken and egg problem is often referred to as Simultaneous Localization and Mapping (SLAM) or Concurrent Mapping and Localization (CML) [1,2], where the robot creates an environmental map, while localizing itself within that map at the same time. The robot has to maintain two kinds of representations concurrently: one is an environment observation model or map, the other is a localization model or position estimate. That means to execute them iteratively, with the output of one being the input of the other [3]. The robot must also be able to run two perceptual procedures, namely map-building and robotic self-localization [4,5]. This phenomenon is depicted in Figure 1.

In the research area of computer vision, landmark extraction is the preliminary step of many vision tasks such as object tracking, localization, recognition and mapping. Manufactured environments are typically composed of easy to recognize geometric features (planes, lines, corners, etc.), but in subsea environments usually no such particular distinguishable features can be found. Considering the restrictions of underwater perception and localization sensors, which are affected by accumulated uncertainty over long-term manipulations, the implementation of robotic underwater SLAM-based navigation is still a tough research topic. Moreover, acoustic sensors for underwater mapping usually provide noisy and distorted images or low-resolution ranging, while video sensors could return highly detailed images but are always limited by water turbidity and poor illumination conditions [5,6].

There are several types of sensors that can be used to obtain distance measurements, such as laser sensors, infrared sensors, digital cameras, and Sound Navigation And Ranging (SONAR) sensors. In our previous works [7,8], we focused on determining underwater geometric features with sonar sensing. Sonar sensors have been widely used in autonomous mobile vehicles, since sound propagates larger distances than electromagnetic waves, sonar imaging outperforms optical imaging in underwater. Especially the side-scan-sonars (SSSs) are increasingly applied in industry and academic research activities to survey the sea floor [9]. High-resolution SSSs create a 2D photorealistic image of the surveyed seafloor. As long as the sonar scans are incorporated through mosaicking, we could recognize the detected underwater objects easily and these feature interpretation offers valuable insights into the study of the seabed topography [10]. The SSSs create higher qualitative sonar images than the forward-looking sonars (FLSs), since SSSs scan the seafloor at a small grazing angle. Generally, the SSS and the FLS could generate large scale maps on the area of the seabed, which are typically processed for obstacle detection and feature extraction [11]. The sonar images with high resolutions are of crucial importance in decision-making to investigate a detected target or not. Several approaches tackle the issues related to object detection and classification, image segmentation and registration, and map fusion.

The following section of this paper introduces the related works about the challenges and the current solutions for the robotic underwater SLAM-based navigation problem. In Section 3, several kinds of state of the art map representations in autonomous robot navigation systems are presented. Once compared, the landmark map is the one that is most qualified to describe the underwater environment. The mathematical model of our presented AEKF-based underwater SLAM algorithm is illustrated in Section 4. It stores the robot pose and the map landmarks in a single system state vector, and estimates the state parameters via a recursive, iterative, three-stage procedure comprising a prediction, an update stage (as in conventional EKF) and a new augmentation stage. Section 5 demonstrates the dense loop mapping and line mapping experiments executed by AEFK-SLAM and FastSLAM 2.0 approaches. The simulations are performed in the context of a mobile robot with a range-bearing sensor in an underwater 2D area. What’s more, the further validation of our proposed underwater AEKF-SLAM algorithm in the SWARMs project, especially the seabed mapping use case is introduced. Section 6 concludes this paper and discusses future works like employing the AEKF approach to manage practical, large-scale robotic underwater navigation problem.

## 2. Related Works

Since radio waves cannot penetrate water very far, an AUV loses its Global Positioning System (GPS) signal as soon as it is diving. Therefore, a standard solution for AUVs to navigate below the water superficies is through dead reckoning. However, navigation can be improved by employing an acoustic underwater positioning system. For underwater robotics, the Inertial Measurement Unit (IMU) and the Doppler Velocity Log (DVL) sensors are most commonly applied for measuring the navigation data on AUVs. While acoustic sensors, such as side scan sonar (SSS) or imaging sonar are used to detect the environmental information. Currently, the fusion of data measurements derived from various perception and on-board navigation sensors plays an important role in robotic underwater mapping researches. In general, we employ acoustic sensors to build large-scale maps of the exploring areas, and optical sensors to return much higher quality images of the targets of interest. To a large extent, the navigation time of the AUV greatly influences the expense of subsea mapping, thus any enhancement in the characteristics of sonar images is of vital concern to the marine and offshore research community.

### 2.1. Solutions to the Underwater SLAM Problem

The research of estimation approaches for the robotic SLAM-based navigation problem has caught utmost attention by the research community. Among the solutions presented in the literature to solve the underwater SLAM problem, the Bayesian filters-based estimation methods have been the most successful ones over time [12,13,14]. Initial works such as [15,16] built a statistical basis for illustrating relationships between landmarks and manipulating geometric uncertainty. At the same time, [17,18] considered employing the Kalman Filter (KF)-based algorithms for visual robotic navigation. After that, [19] demonstrated that, when a mobile robot explores an unknown region observing relative measurements of landmarks, the estimates of these landmark positions are all necessarily correlated with each other, due to the common uncertainty in the estimated robot pose. The SLAM-based robotic navigation problem can be categorized into various solutions, relying on the number of features, the area of coverage, computational complexity, flexibility, reliability, etc. [20]. A general classification of the state of the art, i.e., recursive Bayesian filters-based estimation strategies, including Kalman Filter (KF), Extended Kalman Filter (EKF), Expectation Maximization (EM), Particle Filter (PF), Compressed Extended Kalman Filter (CEKF) and Information Filter (IF) in underwater applications is given, contrasting the advantages and disadvantages summarized in Table 1. The Stochastic SLAM is subject to three dominating defects: high computational load and storage costs, fragile and even wrong data association, and inconsistent update of non-linearity. Keeping relationships between the robot poses and all correlated landmark positions in the whole system state covariance matrix, leads to high computational requirements and space consumption problem. Although some approaches have been proposed to deal with the underwater environment, the SLAM applications for it still remain unsolved.

Table 2 resumes the current popular underwater SLAM methods. Many successful applications exist in the literature employing an EKF to solve nonlinear estimation problems, such as robotic localization, object tracking and it remains to be a popular choice for the solution to the robotic SLAM problems [21].

However, the quadratic computational complexity of the EKF makes it difficult to apply in underwater scenarios. Unscented Kalman Filter (UKF) [35] is a more reliable estimator than EKF when the system model is highly nonlinear, since it approximates the probability density function instead of the nonlinear function itself, but it does not make any improvement in the high computational load of the EKF. The Hector SLAM algorithm is based on a Gauss-Newton approach and replies on scan matching alone to estimate the pose. It is only suitable for few limited unstructured environments, such as the Urban Search and Rescue (USAR) scenarios [36]. Another state of the art solution is GMapping, based on Rao-Blackwellized particle filter (RBPF), which uses odometry for pose estimation [37]. Hector SLAM and GMapping are two laser scanner based SLAM algorithms, and they create an occupancy grid map from laser scans. However, laser range finders are imprecise working in subsea due to light attenuation. Besides, occupancy grid SLAM can only work in indoor environments over a limited period and it cannot deal with an appropriate uncertainty model, so it would diverge in the long-term navigation required under water. Therefore, both Hector SLAM and GMapping are not adequate in our considered case of underwater environments.

In 2002, Montemerlo et al. proposed the classic FastSLAM algorithm [38], which utilizes the particle filters and handles the computational complexity considerably well compared to EKF and UKF. In FastSLAM version 1.0, only the control signal is adopted to sample the new robot pose for each particle according to the robotic motion model, however FastSLAM version 2.0 not only utilizes the robotic control signal but also the sensor measurements together to sample the new robot pose. Thus, FastSLAM 2.0 requires fewer particles and is more robust and accurate than FastSLAM 1.0, besides it will be particularly efficient if an accurate sensor is employed. Although the classical FastSLAM 2.0 algorithm is not a new approach, it has been widely used in many robotic applications, such as robot path planning. In this paper, a new solution, the AEKF-based SLAM algorithm is presented, and we will compare the performances in terms of accuracy of robotic localization and landmarks mapping. Also the computational cost of the FastSLAM 2.0 is compared to that of our proposed AEKF-SLAM algorithm.

### 2.2. Challenges in Underwater SLAM

Nowadays, large-scale dynamic environments still present various challenging problems for the robotic underwater SLAM-based navigation applications due to the following reasons:

#### 2.2.1. Sensors

Generally, the data observed by sonar sensors has limited accuracy, usually with a high angular uncertainty of 22.5% to 30% [39], particularly in environments with low-light, strong ocean currents and turbid waters. Also, the sensors usually only allow limited depth for operation, making applications costly for deep immersions. The estimated noise ends up causing crucial impact on the tasks of localization and mapping, often leading to a non-convergent system. As a consequence, a new calibration is required to obtain a better estimation of the system.

#### 2.2.2. Feature Extraction

In the underwater SLAM context, as many as possible distinguishable features must be observed repeatedly, in order to decrease the uncertainty caused by significant vehicle drift. Here, distinct landmarks can simplify the data association process of fusing new observations to corresponding map features, already stored in the map. In man-made environments, typically composed of planes, lines, corners and points, features can be defined easily; but there are no similar objects or particular features and located easily distinguishable in complex subsea environments. The main problems are the velocity of sound in the water, turbidity, occlusions, suspense material in the water column, seafloor reflection, and surface sonar altitude corrections. Moreover, we need to manage reflections and poor resolutions of the derived acoustic imageries for feature extraction. Also, the dynamic continuous change of underwater natural resources is another factor that makes the recognition of previously visited locations difficult or even impossible.

#### 2.2.3. Absolute Location

Since the underwater environment does not allow using the Global Positioning System (GPS), alternative solutions such as triangulation systems Long Base Line (LBL), Short Base Line (SBL), and Ultra Short Base Line (USBL) have been provided. When operating within a net of sea floor deployed baseline transponders, this is known as LBL navigation. When a surface reference such as a support ship is available, SBL or USBL positioning will be used to calculate where the subsea vehicle is relative to the known GPS position of the surface craft by means of acoustic range and bearing measurements. However, these systems require great efforts for installation, a special demand of logistics, and high costs. Besides, the above solutions limit the working area of robotic operations.

#### 2.2.4. Computational Complexity

The computational requirements of SLAM applications are closely related with the size of the exploring environment and the methods used for feature extraction, tracking, data association, and filtering. The uncertainties of the robot and landmark positions and their correlations will become larger as the number of map elements increases.

## 3. Map Representations

Learning maps is one of the most fundamental and important problems in mobile robotics, since successful robotic systems depend on geological maps that demonstrate the surrounding environment of the robot. Maps are necessary for robotic path planning and self-positioning with respect to the environment, in order to prevent collisions. The basic functionalities of the three most commonly used and popular map representations are summarized in this part and a survey of their suitability to a priori map localization problem is made, such as computational requirement and storage, convergence, robustness, reliability, etc.

### 3.1. Navigational Maps and Their Applications to Underwater SLAM

The occupancy grid maps, the topological maps, and the landmark maps are three kinds of famous and popular map representations in the robotic navigation problem. The suitability for a posterior SLAM estimate problem follows, considering the crucial criteria to establish a tractable and consistent SLAM algorithm. These criterions are illustrated as follows, note that the first two are only valid for metric maps. Taking into account the sparse spatial distribution of underwater features, and the far distances between distinct features, landmark maps are most suitable to represent subaquatic areas.

#### 3.1.1. Representation of Uncertainty

Since mobile vehicle sensors cannot measure positions in the environment with total accuracy, a degree of uncertainty should be used in map representations. Meanwhile, the vehicle position is acquired from this map, so the pose estimate is also uncertain. This paper specifies that both, environmental map and robot pose estimates, require an appropriate quantitative uncertainty, and that uncertainty model is able to reflect the error between the estimated and the actual system state.

#### 3.1.2. Monotonic Convergence

The main purpose of an uncertainty measurement is to ensure map convergence. A convergent map is given if the estimated environmental geometry is equal to true physical geometry when new observation data is incorporated. In other words, the map uncertainty decreases monotonically. Without this uncertainty model, a stationary object with estimated position $\left(x_{1},y_{1}\right)$ may drift to some arbitrarily distant place $\left(x_{2},y_{2}\right)$, with subsequent map updates. Therefore, explicit uncertainty is needed to assess map accuracy and it constrains the influence of subsequent observation information.

#### 3.1.3. Data Association

The map representations should permit reliable correspondence between the measurements derived from the robot sensors and the information gained from the stored map. First of all, the observation-to-map data association should be efficient enough for real-time operation. Secondly, the data association needs to be robust enough for partial views and large-scale searching areas, since an observation is composed of a combination of the currently mapped area, unexplored regions and dynamic features. Due to the size of the exploring area is determined by the robot pose uncertainty, a precise uncertainty model could enhance both robustness and efficiency, by stipulating a minimal detecting region.

#### 3.1.4. Loop Closure

When a mobile robot explores a working environment by navigating in a large cycle that is much bigger than its sensing range, then return and recognition of an already mapped area is called the “Loop Closure Problem”. Other denominations are “Cycle Detection Problem” or “Map Revisitation Problem”. Loop closure mainly considers two previous criterions: one is the data association, which is different from local association as a result of the much larger robot pose uncertainty and the related search space. Exploring efficiency is one important aspect, but it is more crucial to decide if an association is correct or an artifact of environmental similarity should be robustly stable. The other issue, after associating sensor measurements correctly, is the convergence problem, where a long-term accumulated error in the map loop must be compensated properly during the map update stage by propagating the error-offset back through the map cycle.

#### 3.1.5. Computational and Storage

The map has to store substantial information for enabling data association and convergence. The computation and the storage required to update the map with newly observed measurements, must be scaled reasonably according to the detected environmental region.

### 3.2. The Occupancy Grid Maps

The 2D occupancy grid maps, which are also known as evidence grid maps were introduced by Moravec [40]. In the occupancy grid maps, the environment is represented in a discrete grid composed of rectangular cells with the same shape and size. Each cell is assigned a value representing the probability of occupancy. A probability of 1 means that the cell is definitely occupied and the robot cannot pass through it. If it is 0, the cell is definitely vacant, and the robot can traverse it. An occupancy probability of 0.5 declares an unknown state. The larger the cell value is, the more the cell tends to be occupied; the smaller its value is, the more the cell tends to be free. Usually in the occupancy grid maps, black cells imply occupied areas, white cells stand for empty regions and the gray ones mean unexplored spaces.

The occupancy grid SLAM interleaves the localization and map update stages by first registering the short-term local map with the global map (localization), and then updating the perceived occupancy of the global map grid cells (map building). In dynamic indoor areas, the occupancy grid map works efficiently during a limited exploring time. The occupancy grid maps are adequate for local navigation and obstacle avoidance purposes [41] and thereby it is popular to describe the environment of a mobile robot, given known poses.

Nevertheless, the occupancy grids do not process an appropriate uncertainty model, and so they will diverge in the long-term operations. The main drawback of the occupancy grid maps is that they do not scale well to large environments, since they can only describe the uncertainty model from the local robotic view. Also, memory size is a limiting factor, when the goal is to globally map a large region. To adequately capture the details in more complex underwater environments, a high resolution of cell distribution is required, which is wasted in less complex areas. Techniques like quad trees or octrees [42] have been presented to deal with the space storage issue, but they also increase the processing burden.

### 3.3. The Topological Maps

**Figure 2:**
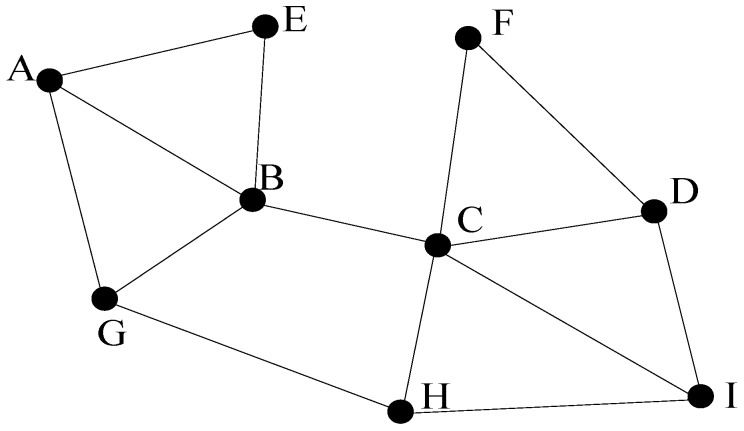
The topological map.

The topological maps do not need metric measurements, since they depict the exploring regions by the paths leaded by the feature locations, as shown in Figure 2. A vertex represents a landmark, such as a particular location in the environment, and an edge indicates the traversability between two connected nodes. Thus, navigation between two non-adjacent places is determined by a sequence of transitions between intermediate location nodes, and so standard graph shortest path algorithms can be used (e.g., A to D requires travelling through the sequence A→B→C→D). The concept works on the assumptions that distinctive places are locally distinguishable from the surrounding area, and the procedural information is sufficient to enable the robot to travel within the recognizing distance of a specified place [38].

The efficient topological maps have compact representation form, thus they are suitable for robotic navigation and fast path planning tasks. The departure from metric representation makes pose uncertainty estimation irrelevant, and qualitative measurements are used like “follow the path from A to B” or “at B” instead. The ability to use standard graph algorithms for high-level planning operations, such as the shortest path search between two non-adjacent nodes, is of particular advantage.

Nevertheless, without some form of metric position measurement, the significant drawback of topological maps is that they cannot ensure reliable navigation between distinct places, and subsequent feature recognition. Although the relationship between different places is maintained, distance and direction are subject to change and variation. The employment of purely qualitative trajectory information, such as wall following [43] to travel between distinguishable landmarks, is suitable for static structured environments, but may guide the robot to an improper vicinity of the right place in more complex and dynamic environments. Essentially, unless the exploring space possesses at least one globally unique sequence of landmark positions, loop closure must always be ambiguous. This is the crucial weakness in the topological map paradigm, since underwater landmark similarities may eventually generate a consistently similar sequence of places and results in wrong data association. The solution to this problem is to introduce some form of metric information, which would enable the estimation of pose uncertainty between landmarks. By bounding the cycle exploring space, place sequences only need to be locally unique.

In our underwater scenario, where position identification is quite complicated, the probability of wrong localization is high [44]. Due to the lack of exact physical measurements in the seabed, the topological maps are not adequate for robotic underwater SLAM-based navigations.

### 3.4. The Landmarks Maps

**Figure 3:**
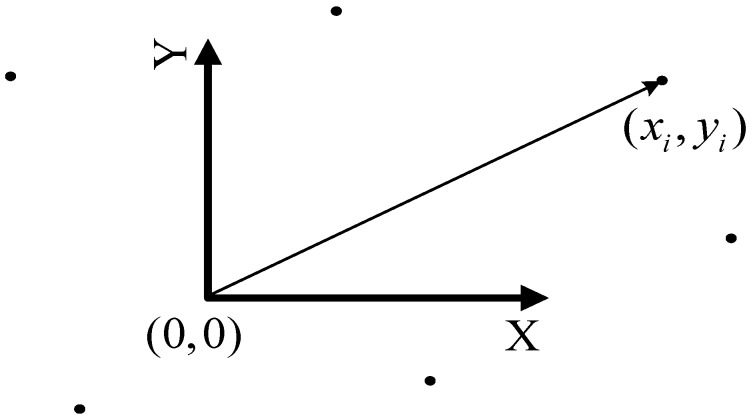
The landmark map.

Landmark maps, also called as the feature maps, use geometric primitives such as corners, points, and lines to describe the working space, as shown in Figure 3. The features can be artificial landmarks, natural landmarks [45], and they can be of abstract form, detected by certain algorithms [46,47]. Localization is performed by extracting features from sensor measurements and associating them to other features that are already stored in the map. Then, the differences between the predicted feature locations and the detected positions are used to calculate the robot pose by the estimation filters. In this way, localization is very like a multiple target tracking problem [48], but here the targets are static and the observer is in motion.

Recursive EKF pose estimation has the advantages of efficient data fusion from multiple sensor measurements and the ability to incorporate explicit sensor uncertainty models. Besides, the memory size needed to store a landmark map is very small compared to an occupancy grid map or a 3D map, and it has high flexibility in map adjustment. In a 2D landmark-based map, feature positions are stored in the Cartesian coordinate system. A covariance matrix associated with the map is used to describe the uncertainties of both landmark positions and robot poses [34].

Nevertheless, landmark maps have some limitations. First of all, the underwater landmarks have to be extracted from noisy sensor measurements, so it is required to identify features in the noisy observations. Secondly, a correct data association is essential to build consistent landmark-based maps [49]. Incorrect data association will reduce the accuracy of a landmark map and even leads the filter to diverge. A further problem concerning feature maps is that they are only suitable for environments where the observed objects can be reasonably depicted by basic geometric feature models, but the introduction of new geometric primitives would increase the map complexity.

Landmark map-based SLAM comprises the dual task of adding newly detected features to the map using the robot pose as a reference, while applying existing map features to reckon the robot pose iteratively. Therefore, the uncertainty of sensor measurements results in uncertain estimates of both the robot pose and the map feature positions, and these uncertainties are dependent. Correlated uncertainty is of importance to feature-based SLAM since it inevitably couples the individual landmarks to each other and the robot to the map. Attempts to estimate the robot pose and map features independently have been shown to produce inconsistent uncertainty estimates.

In summary, the landmark maps are the most suitable ones to demonstrate the robotic underwater SLAM-based navigation, when observable and tractable landmarks are present. The stationary points, which are the least complex features, are taken into account in this work to describe the landmarks within a local subsea region. This simplification not only decreases the challenges with feature recognition and interpretation, but also increases the focus on our presented AEKF-SLAM algorithm itself. So as to achieve robust and convergent applications in larger marine environments with continuously moving objects, we need to modify the conventional stochastic SLAM approaches.

## 4. The Posterior-Estimated AEKF-SLAM Algorithm

In this part, the mathematical model of our proposed “Robotic underwater AEKF-SLAM based navigation” algorithm is established. Here we adopt the same notation which was employed in [7,19,49].

### 4.1. Vehicle Model

The setting for the SLAM problem is that of a robot with a known kinematic model, starting at an unknown position, and moving through the exploring space containing multiple features. The robot is equipped with sensors to measure the relative location between any detected landmark and the robot itself. The absolute landmark positions are not available. Without previous knowledge, a linear synchronous discrete-time model composed of the evolution of the robot poses and the landmark observations is adopted. Although the robot motion model and the landmark measurements are usually nonlinear and asynchronous in any real navigation application, the use of linear synchronous models does not affect the validity of the proofs in the SLAM problem other than to require the same linearization assumptions as those normally employed in the development of an EKF [38]. Indeed, the implementation of the SLAM algorithm uses a nonlinear robot model and a nonlinear asynchronous observation model. The state of the system of interest consists of the position and orientation of the robot together with the all landmark locations. The robot state at time *k* is indicated by $x_{v}(k)$, and the robot motion is modeled by a conventional linear discrete-time state transition equation:

(1) $$x_{v}(k+1)=F_{v}(k)x_{v}(k)+u_{v}(k+1)+\omega_{v}(k+1)$$

where $F_{v}(k)$: State transition matrix; $u_{v}(k)$: Vector of control inputs; $\omega_{v}(k)$: Vector of temporally uncorrelated procession noise errors; it complies with normal Gaussian distribution, and its covariance matrix is denoted as $Q_{v}(k)$.

### 4.2. Feature Model

The AEKF-SLAM algorithm is based on a landmarks map. Repeatable observation of features is a mandatory requirement for SLAM. This paper considers the least complicated features, which are stationary point landmarks. More elaborate parametric feature models, such as lines, might also be used, but are not implemented in this work.

The *i*th landmark position is defined as $x_{m_{i}}$. Without loss of generality, the number of all landmarks is arbitrarily set to *N*. Since the point feature is assumed to be invariant, the state transition equation for it is:

(2) $$x_{m_{i}}(k+1)=x_{m_{i}}(k)=x_{m_{i}}\;i=1,2,\cdots ,N$$

where the matrix of all *N* landmarks is $X_{m}={\left[x_{m_{1}}^{T},\ldots ,x_{m_{N}}^{T}\right]}^{T}$ with *T* transpose and used both inside and outside the brackets in order to conserve the dimension of space. The augmented state matrix is composed of both, the states of the robot and all landmark positions, and is expressed as:

(3) $$x_{a}(k)\;=\;{\left[x_{v}^{T}(k),x_{m_{1}}^{T},\ldots ,x_{m_{N}}^{T}\right]}^{T}\;$$

Consequently, the augmented state transition model for the complete system can be rewritten as:

(4) $$[\begin{array}{c} x_{v}(k+1)\\ x_{m_{1}}\\ \vdots \\ x_{m_{N}} \end{array}]=[\begin{array}{cccc} F_{v}(k) & 0 & \cdots & 0\\ 0 & I_{x_{m_{1}}} & \cdots & 0\\ \vdots & \vdots & \ddots & 0\\ 0 & 0 & 0 & I_{x_{m_{N}}} \end{array}][\begin{array}{c} x_{v}(k)\\ x_{m_{1}}\\ \vdots \\ x_{m_{N}} \end{array}]+[\begin{array}{c} u_{v}(k+1)\\ 0_{x_{m_{1}}}\\ \vdots \\ 0_{x_{m_{N}}} \end{array}]+[\begin{array}{c} \omega_{v}(k+1)\\ 0_{x_{m_{1}}}\\ \vdots \\ 0_{x_{m_{N}}} \end{array}]$$

or equivalently:

(5) $$x_{a}(k+1)=F_{a}(k)x_{a}(k)+u_{a}(k+1)+\omega_{a}(k+1)$$

where $I_{x_{m_{i}}}$ is the dim(*x_m_i__*) × dim(*x_m_i__*) identity matrix and $0_{x_{m_{i}}}$ is the $\dim(x_{m_{i}})$ null vector.

Actually, any landmark $x_{m_{i}}$ which is in stochastic motion may be easily adapted to this framework. However, doing so offers little insight into the problem and even the convergence properties may not be held [50].

### 4.3. Observation Model

The robot is equipped with sensors to measure observations of the positions of landmarks relative to the robot. We assume that observations are linear and synchronous. The observation model for the *ith* landmark can be denoted in the following form:

(6) $$\begin{array}{ll} z_{i}(k) & =H_{i}x_{a}(k)+\upsilon_{i}(k)\\ & =H_{x_{m_{i}}}x_{m}-H_{v}x_{v}(k)+\upsilon_{i}(k) \end{array}$$

where $v_{i}(k)$ is a vector of temporally uncorrelated observation errors and its covariance matrix is denoted as $R_{i}(k)$. The observation matrix $H_{i}$ relates the sensor outputs $z_{i}(k)$ to the state vector $x_{a}(k)$ when detecting the ith landmark. Note that the observation model for the *i*th landmark has the following form:

(7) $$H_{i}=[-H_{v},0,\ldots ,0,H_{x_{m_{i}}},0,\ldots ,0]=[-H_{v},H_{m_{i}}]$$

This structure implies that the observations between the robot and the landmarks are often in the form of a relative position, or relative range and bearing.

### 4.4. Simultaneous Localization and Mapping

**Figure 4:**
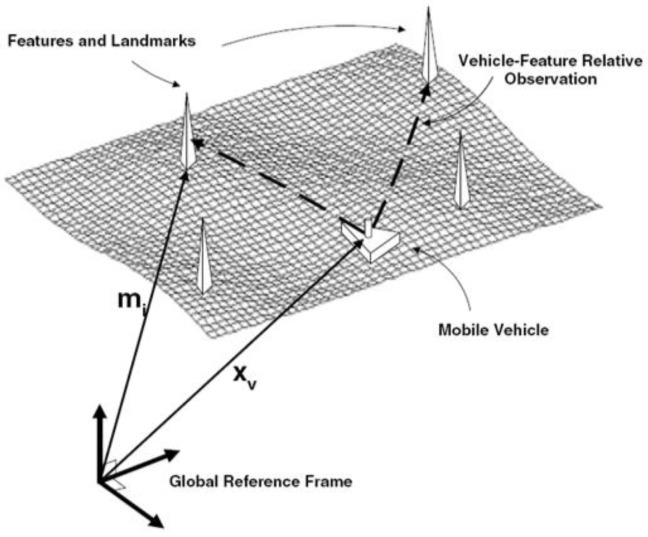
A robot measuring relative observations to environmental landmarks.

Robotic SLAM-based navigation integrates the Received Signal Strength Indication (RSSI) samples derived from the detected targets with the data recorded by the robotic odometers to build and refine the exploring environmental map, while concurrently localize the robot itself in this map [51,52,53]. If the robot’s path were known with certainty, then mapping would be a straightforward problem. The landmark locations in the robot’s surrounding could be estimated by using independent filters. Nevertheless, in the SLAM problem, the robotic trajectory is unknown, thus the uncertainties of the robot poses can be arbitrarily large due to the accumulated odometry errors, which also leads to errors in the robot path correlating errors in the map. Therefore, the state of the robot and the map features must be estimated at the same time. The structure of landmark-based SLAM is shown in Figure 4.

#### 4.4.1. The SLAM Process

**Figure 5:**
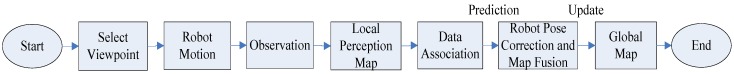
The flowchart of the SLAM process.

The general SLAM process is illustrated in Figure 5. Each time, after sensor measurements, the robotic local perception map needs to be integrated with the global view map in order to update the robot pose and also refine the detected landmark coordinates. The challenge of the robotic SLAM-based navigation problem is that accurate robot poses are needed to generate a qualified map. Nevertheless, when the unlimited incremental odometry errors are lowered, sensor measurements need to be incorporated into a precise map. SLAM implies a set of difficulties, such as the correct associate sensor measurements, an efficient mapping of large-scale environments, and the robust prediction of the robot path.

**Figure 6:**
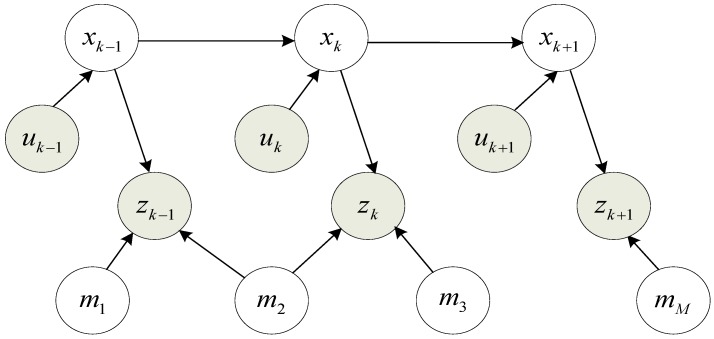
The SLAM graphical model.

The SLAM problem is depicted as Bayes network in Figure 6 to understand the dependencies in the SLAM problem. The figure shows the changes of robot poses from $x_{k-1}$ to $x_{k}$ by receiving the control signal $u_{k}$, the observation $z_{k-1}$, the robot pose $x_{k}$ and the landmarks $m_{M}$. The arrows show direct dependencies, while there is no direct relationship between robot poses and landmarks [54]. Shaded nodes represent data directly observable by the robot, SLAM is that the robot recovers the unobservable variables-landmarks.

#### 4.4.2. Loop Closing

**Figure 7:**
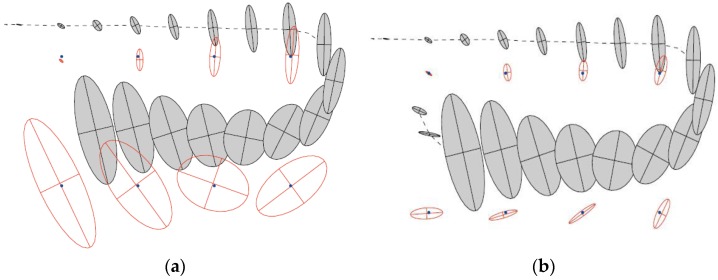
(**a**) Before closing the loop; (**b**) After closing the loop.

Tracked landmarks could provide a basis for reducing the uncertainty of the robot poses. In closed loops, if a robot can detect a position where it has been before and could correctly match landmarks, then the accumulated errors will be bounded and the map precision will be increased [55,56,57]. The correlation between the robot pose uncertainty and map uncertainty is shown graphically in Figure 7a. The robot is moving along the path drawn as the dashed line, observing nearby eight distinguishable landmarks, drawn as dots. The shaded ellipses imply the uncertainties of the robot about its own poses, drawn over time. As a result of the control error, the robot pose becomes more uncertain when the robot moves. The estimations of the landmark positions are specified by white ellipses. One can see that as the robot pose becomes more uncertain, the uncertainty in the estimated locations of newly observed landmarks also increases.

In Figure 7b, the robot completes the loop and revisits a previously observed landmark. As the coordinate of the first observed landmark has high precision, the uncertainty of the predicted robot pose is significantly reduced. Therefore, also the position uncertainties of the previously perceived landmarks decrease. The resulting effect is that the information spreads to previously observed landmarks, such that gaining information on the robot pose is probably the most important characteristic of the posterior SLAM estimate [58]. In Figure 7b, it can be seen that the shaded ellipses obtained before loop closure do not shrink after closure, because they depict a time series of robot pose uncertainties and are not revised afterwards.

The ability of re-identifying previously detected features are of crucial importance to the cycle detection problem, since robot revisitation enhances the accuracy of robotic localization and landmarks mapping. As a consequence, we present the AEKF-SLAM based robotic navigation algorithm to identify each sensor perception as a new landmark or a previously observed one.

### 4.5. Augmented Extended Kalman Filter

Practically an EKF rather than a simple linear KF is employed to generate state estimates. Although the EKF suffers from high computational complexity, it has the highest convergence among the current methodologies. It has been successfully and widely employed in large-scale environments, including land, air and even underwater [59]. For the underwater nonlinear discrete SLAM estimation problem, although the linearization errors of the conventional EKF decrease the localization accuracy, EKF generally produces satisfying performances due to its straightforward conception and relatively low computational complexity. The standard solution to manage the nonlinear SLAM system is to linearize the robotic kinematic model and the landmark observation model by an EKF for generating the system state predictions. It is supposed that the nonlinear discrete system has the following form:

State function

(8) $$\text{State function }f(\cdot ):X_{k}=f(X_{k-1})+\varpi_{k}$$

(9) $$\text{Observation function }h(\cdot ):Z_{k}=h(X_{k})+v_{k}$$

where $\varpi_{k}$ is the procession noise and obeys the standard Gaussian distribution $\varpi_{k}∼N(0,Q_{k})$, $Q_{k}$ is its covariance matrix; $v_{k}$ is the observation errors and complies with the standard normal distribution $v_{k}∼N(0,R_{k})$, $R_{k}$ is its covariance. As for the AEKF estimator, it mainly consists of three stages, which include state prediction, observation, measurement prediction, matching and estimation [60]. In the prediction stage, the command signal $U_{k}$ and the robot motion model are utilized to estimate the robot pose. Then, in the update stage, to update the landmark positions and to refine the estimation of the robot pose, the new observation $Z_{k}$ from an exteroceptive sensor is used. When a landmark is detected for the first time, however, it is added to the system state vector through an initialization process called state augmentation.

**Figure 8:**
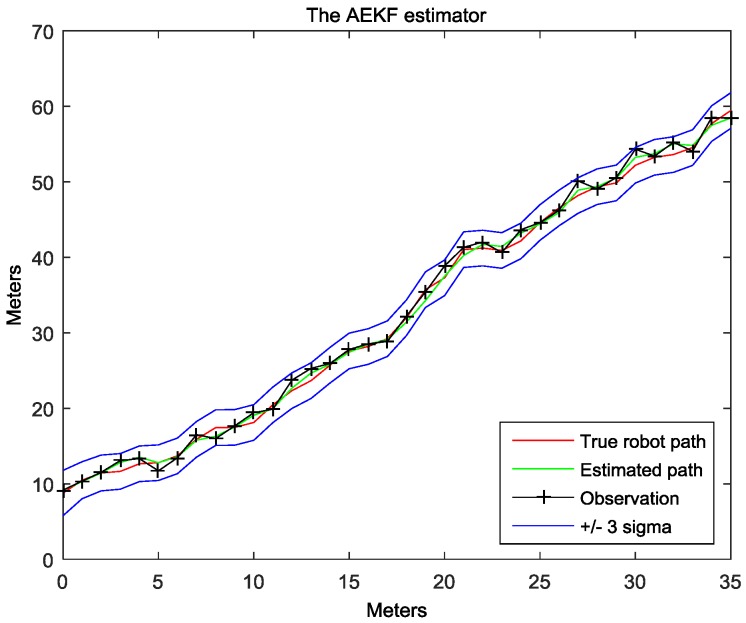
The AEKF estimator.

The simulation of the AEKF estimator is shown in Figure 8. Here we suppose that both, the robot control noise and the sensor observation noise, are equal to 1. And we assign the robot’s start position at the coordinate (0, 7 m) with a velocity of 1 m/s. The true robot path is depicted as the red line, the green line stands for the robot path estimated by the AEKF. The observations are drawn as black line with black crosses ‘+’, which are the surrounding landmarks. The two blue lines mean the +3 sigma and −3 sigma around the true robot path. It proves that AEKF could estimate the robot pose and the landmark positions accurately and robustly.

### 4.6. The Estimation Process of the AEKF-SLAM

As for the popular FastSLAM algorithm [38], which employs the Rao-Blackwellised method for particle filtering (RBPF), is based on an extract factorization of the posterior into a product of conditional landmark distributions and a distribution over robot paths. The FastSLAM behaves much better than the EKF-SLAM at handling the data association issue for the nonlinear system map revisitation. However, the biased noises resulting from the unequal wheels misalignment deviate the robot path to one side, but the classical solutions for the SLAM problem, like FastSLAM or EKF, cannot estimate precisely, since they suppose zero mean noise while compensating odometry errors. As a result, here we present the AEKF-SLAM based algorithm to deal with the robotic underwater SLAM problem. The experiments performed later in this paper show that the AEKF-SLAM approach builds a more accurate landmark map and also estimates the robot trajectory more precisely than the FastSLAM 2.0.

**Figure 9:**
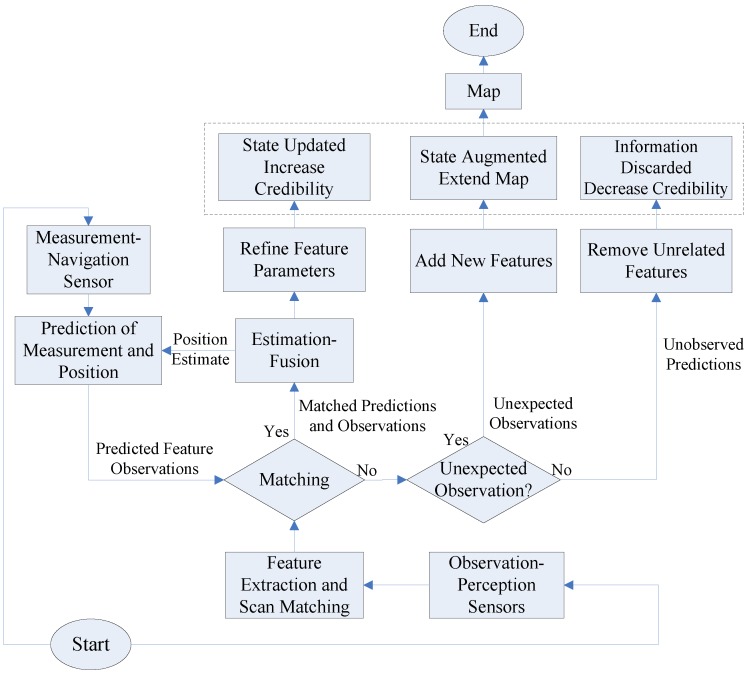
The flow chart of SLAM procedure based on an AEKF, modified in [7].

In any SLAM algorithm, the position and number of environmental landmarks is not known a priori, landmark coordinates must be initialized and inferred from the sensor observations alone. The AEKF-based SLAM algorithm applies the AEKF to online SLAM by using the maximum likelihood data association method for the correspondence test of features. Here, the recursive AEKF-SLAM based robotic navigation algorithm, includes a prediction phase, an observation phase (like in traditional EKF), and additionally a new augmentation. The prediction state estimates the current robot pose using its odometers. Once the robot measures the surrounding targets in the update stage, the coordinates of the detected features relative to the robot side are derived. In the following sensor data fusion procedure, the features from the estimated and actual perceived maps are integrated and the deviations between them are applied to track the robot path and refine the detected landmark locations. The sensor measurements consist of the following data: new features, already observed features and observations without any direct relationship. After sensor data association, new features extend the system state, associated features increase the precision of the system, and unrelated features are rejected. The overall procedures of the AEKF-SLAM based robotic navigation algorithm are illustrated in Figure 9.

Table 3 summarizes the necessary procedures of the presented algorithm of the AEKF-SLAM based robotic underwater navigation. As soon as the feature extraction and the data association are in place, the AEKF-SLAM method can be considered as the following three steps. First and foremost, predict the robot current state using the odometry data. Next, update the estimated state from re-observed landmark positions. Eventually, add new detected landmarks in the map. If a feature is perceived for the first time, then it is included to the system state by the proposed augmentation state.

**Figure 10:**
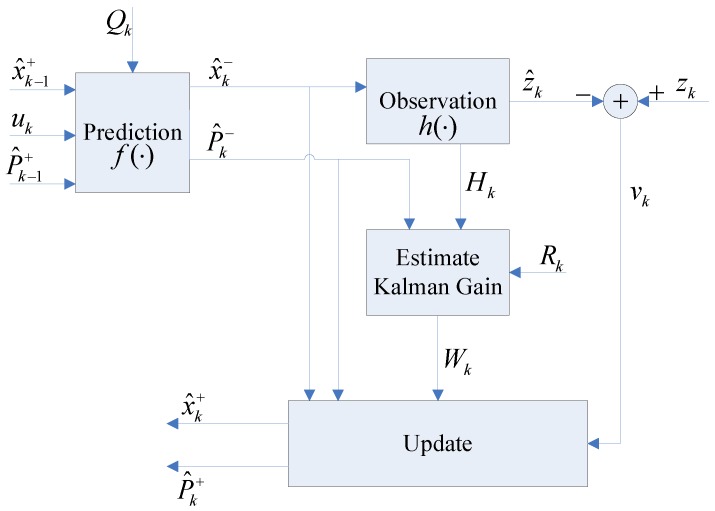
The architecture of the AEKF-SLAM-based robotic navigation system, as in [7].

The architecture of our presented AEKF-SLAM based robotic navigation system is demonstrated in Figure 10. ${\hat{X}}_{k}$ and ${\hat{P}}_{k}$ are the predicted system state and its covariance matrix. The filter iteratively refines the state mean ${\hat{X}}_{k}^{+}$ and state covariance ${\hat{P}}_{k}^{+}$ through integrating the estimated state mean ${\hat{X}}_{k}^{-}$ and state covariance ${\hat{P}}_{k}^{-}$ with the new perception $z_{k}$. $Q_{k}$ and $R_{k}$ are the covariances of procession noises and observation errors, separately.

**Algorithm 1.** AEKF-SLAM-based robotic underwater navigation.For k=1 to N$[X_{k}^{-},P_{k}^{-}]=Predict(X_{k-1},P_{k-1});$$z_{k}=Get\;Observation();$$[z_{o},z_{n}]=Data\;Association(X_{k}^{-},P_{k}^{-},z_{k},R_{k});$$[X_{k}^{+},P_{k}^{+}]=Update\;Map(X_{k}^{-},P_{k}^{-},z_{o},R_{k});$$[X_{k}^{+},P_{k}^{+}]=Augment\;Map(X_{k}^{-},P_{k}^{-},z_{n},R_{k});$End for

The pseudo code of our presented AEKF-SLAM solution for robotic underwater localization and mapping is summarized in Algorithm 1 above. Where $z_{o}$ stands for the previously observed features, $z_{n}$ represent newly detected landmarks. Varying bathymetric height of the AUV makes it tough to build the underwater map in the same scale and identical resolution. Therefore, we assume the practical 3D spatial geometry to be perpendicular to the horizontal plane where the AUV navigates, and we can describe the environment by a simplified 2D model in order to put more attention to our presented AEKF-SLAM algorithm. The fundamental formulas for the AEKF-SLAM based robotic underwater navigation approach are presented as follows.

#### 4.6.1. Vehicle, Map and Augmented State Vectors

The robot state is described by its coordinate and heading angle as:

(10) $${\hat{X}}_{v}={\left[{\hat{x}}_{v},{\hat{y}}_{v},{\hat{\varphi }}_{v}\right]}^{T}$$

with its covariance $P_{v}$:

(11) $$P_{v}=[\begin{array}{ccc} {\sigma^{2}}_{x_{v}x_{v}} & {\sigma^{2}}_{x_{v}y_{v}} & {\sigma^{2}}_{x_{v}\varphi_{v}}\\ {\sigma^{2}}_{x_{v}y_{v}} & {\sigma^{2}}_{y_{v}y_{v}} & {\sigma^{2}}_{y_{v}\varphi_{v}}\\ {\sigma^{2}}_{x_{v}\varphi_{v}} & {\sigma^{2}}_{y_{v}\varphi_{v}} & {\sigma^{2}}_{\varphi_{v}\varphi_{v}} \end{array}]$$

The 2D point landmarks observed by the robot to form a map are in the same base coordinate system as the robot. The coordinate of the *n*th feature is denoted as $x_{m_{n}}=\;{\left({\hat{x}}_{n},{\hat{y}}_{n}\right)}^{T}$, and the environmental landmarks can be described as:

(12) $${\hat{X}}_{m}={\left[{\hat{x}}_{1},{\hat{y}}_{1},\ldots ,{\hat{x}}_{n},{\hat{y}}_{n}\right]}^{T}$$

and its covariance matrix $P_{m}$ is:

(13) $$P_{m}=[\begin{array}{ccccc} {\sigma^{2}}_{x_{1}x_{1}} & {\sigma^{2}}_{x_{1}y_{1}} & \cdots & {\sigma^{2}}_{x_{1}x_{n}} & {\sigma^{2}}_{x_{1}y_{n}}\\ {\sigma^{2}}_{x_{1}y_{1}} & {\sigma^{2}}_{y_{1}y_{1}} & \cdots & {\sigma^{2}}_{y_{1}x_{n}} & {\sigma^{2}}_{y_{1}y_{n}}\\ \vdots & \vdots & \ddots & \vdots & \vdots \\ {\sigma^{2}}_{x_{1}x_{n}} & {\sigma^{2}}_{y_{1}x_{n}} & \cdots & {\sigma^{2}}_{x_{n}x_{n}} & {\sigma^{2}}_{x_{n}y_{n}}\\ {\sigma^{2}}_{x_{1}y_{n}} & {\sigma^{2}}_{y_{1}y_{n}} & \cdots & {\sigma^{2}}_{x_{n}y_{n}} & {\sigma^{2}}_{y_{n}y_{n}} \end{array}]$$

The off-diagonal terms of the covariance matrix *P_m_* are the cross-correlation information between different landmarks. They capture the dependence of each landmark position upon knowledge of the other landmarks in the map. Since the landmarks are assumed to be stationary and their positions do not change over time, these correlations will be enhanced with each re-observation, which makes the map increasingly rigid.

The AEKF-SLAM landmark map is represented by an augmented state vector ${\hat{X}}_{a}$, which is made up of all previously detected landmark locations ${\hat{X}}_{m}$ and the present robot state ${\hat{X}}_{v}$ [7,61]. The cross covariance between the robot pose and environmental landmarks is denoted as $P_{vm}$:

(14) $${\hat{X}}_{a}=[\begin{array}{c} {\hat{X}}_{v}\\ {\hat{X}}_{m} \end{array}],\;P_{a}=[\begin{array}{cc} P_{v} & P_{vm}\\ P_{vm}^{T} & P_{m} \end{array}]$$

Usually, the original conditions of the state estimate are ${\hat{X}}_{a}={\hat{X}}_{v}=0$ and $P_{a}=P_{v}=\;0$, meaning that the robot has not perceived any features until now and the base coordinate system is build upon the initial robot pose.

#### 4.6.2. Prediction Stage

**Figure 11:**
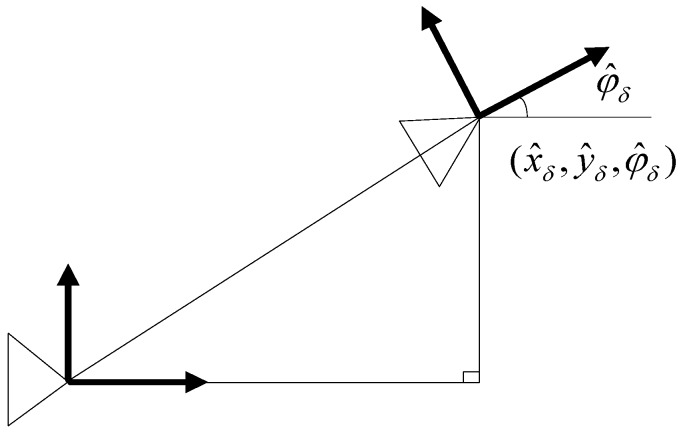
The robot motion model.

The SLAM process model specifies that a robot moves relative to its previous pose according to a dead reckoning motion estimate, and the surrounding landmarks remain still. The effect of this model on the system state estimate is a change in the ${\hat{X}}_{v}$ term of the state vector, and in the $P_{v}$ and $P_{vm}$ portions of the state covariance matrix, however ${\hat{X}}_{m}$ and $P_{m}$ keep constant. An estimate of the underwater robot pose change ${\hat{X}}_{\delta }={\left[{\hat{x}}_{\delta },{\hat{y}}_{\delta },{\hat{\varphi }}_{\delta }\right]}^{T}$ with covariance $P_{\delta }$ (see Figure 11) is commonly obtained by the inertial navigation system (INS) and a robot kinematic model.

As a result, the estimated system state ${\hat{X}}_{a}^{-}$ is calculated as:

(15) $${\hat{X}}_{a}^{-}=f({\hat{X}}_{a},{\hat{X}}_{\delta })=[\begin{array}{c} g({\hat{X}}_{v},{\hat{X}}_{\delta })\\ {\hat{X}}_{m} \end{array}]=[\begin{array}{c} {\hat{x}}_{v}+{\hat{x}}_{\delta }\cos{\hat{\varphi }}_{v}-{\hat{y}}_{\delta }\sin{\hat{\varphi }}_{v}\\ {\hat{y}}_{v}+{\hat{x}}_{\delta }\sin{\hat{\varphi }}_{v}+{\hat{y}}_{\delta }\cos{\hat{\varphi }}_{v}\\ {\hat{\varphi }}_{v}+{\hat{\varphi }}_{\delta }\\ {\hat{X}}_{m} \end{array}]$$

and its prediction covariance matrix $P_{a}^{-}$ is:

(16) $$P_{a}^{-}=JP_{a}J^{T}+QP_{\delta }Q^{T}$$

where the Jacobian matrices *J* and *Q* are given by:

(17) $$J={\frac{\partial f}{\partial X_{a}}|}_{\left({\hat{X}}_{a},{\hat{X}}_{\delta }\right)}={[\begin{array}{cc} J_{v} & 0_{vm}\\ 0_{vm}^{T} & I_{m} \end{array}]}_{\left({\hat{X}}_{a},{\hat{X}}_{\delta }\right)},\;Q={\frac{\partial f}{\partial X_{\delta }}|}_{\left({\hat{X}}_{a},{\hat{X}}_{\delta }\right)}={[\begin{array}{c} Q_{v}\\ 0_{vm}^{T} \end{array}]}_{\left({\hat{X}}_{a},{\hat{X}}_{\delta }\right)}$$

Here, $J_{v}$ and $Q_{v}$ are the Jacobian matrices of partial derivatives of the nonlinear motion function *g* in terms of the current robot state $X_{v}$ and the robot pose change $X_{\delta }$:

(18) $$J_{v}={\frac{\partial g}{\partial X_{v}}|}_{\left({\overset{\wedge }{X}}_{v},{\overset{\wedge }{X}}_{\delta }\right)}=[\begin{array}{ccc} \frac{\partial g_{1}}{\partial x_{v}} & \frac{\partial g_{1}}{\partial y_{v}} & \frac{\partial g_{1}}{\partial \varphi_{v}}\\ \frac{\partial g_{2}}{\partial x_{v}} & \frac{\partial g_{2}}{\partial y_{v}} & \frac{\partial g_{2}}{\partial \varphi_{v}}\\ \frac{\partial g_{3}}{\partial x_{v}} & \frac{\partial g_{3}}{\partial y_{v}} & \frac{\partial g_{3}}{\partial \varphi_{v}} \end{array}]=[\begin{array}{ccc} 1 & 0 & -{\hat{x}}_{\delta }\sin{\hat{\varphi }}_{v}-{\hat{y}}_{\delta }\cos{\hat{\varphi }}_{v}\\ 0 & 1 & {\hat{x}}_{\delta }\cos{\hat{\varphi }}_{v}-{\hat{y}}_{\delta }\sin{\hat{\varphi }}_{v}\\ 0 & 0 & 1 \end{array}]$$

(19) $$Q_{v}={\frac{\partial g}{\partial X_{\delta }}|}_{\left({\overset{\wedge }{X}}_{v},{\overset{\wedge }{X}}_{\delta }\right)}=[\begin{array}{ccc} \frac{\partial g_{1}}{\partial x_{\delta }} & \frac{\partial g_{1}}{\partial y_{\delta }} & \frac{\partial g_{1}}{\partial \varphi_{\delta }}\\ \frac{\partial g_{2}}{\partial x_{\delta }} & \frac{\partial g_{2}}{\partial y_{\delta }} & \frac{\partial g_{2}}{\partial \varphi_{\delta }}\\ \frac{\partial g_{3}}{\partial x_{\delta }} & \frac{\partial g_{3}}{\partial y_{\delta }} & \frac{\partial g_{3}}{\partial \varphi_{\delta }} \end{array}]=[\begin{array}{ccc} \cos{\hat{\varphi }}_{v} & -\sin{\hat{\varphi }}_{v} & 0\\ \sin{\hat{\varphi }}_{v} & \cos{\hat{\varphi }}_{v} & 0\\ 0 & 0 & 1 \end{array}]$$

These Jacobians matrices only have influences on the robot portion of the covariance $P_{v}$ and its cross-correlated convariance $P_{vm}$, thus the estimated system covariance $P_{a}^{-}$ is computed and implemented more efficiently as:

(20) $$∴P_{a}^{-}=[\begin{array}{cc} J_{v}P_{v}J_{v}^{T}+Q_{v}P_{\delta }Q_{v}^{T} & J_{v}P_{vm}\\ {\left(J_{v}P_{vm}\right)}^{T} & P_{m} \end{array}]$$

#### 4.6.3. Observation Stage

**Figure 12:**
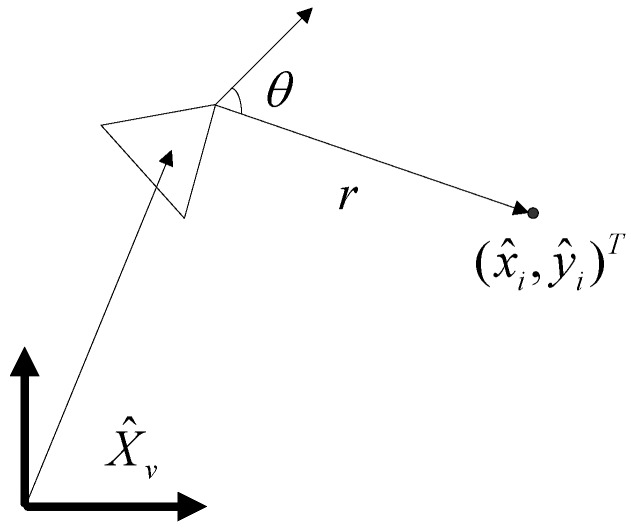
The robot observation model.

We assume that a landmark, which is already stored in the map as an estimate $x_{m_{i}}=\;{\left({\overset{\wedge }{x}}_{i},{\overset{\wedge }{y}}_{i}\right)}^{T}$, is perceived by a range-bearing sonar with the measurement *z* (see Figure 12):

(21) $$z=[\begin{array}{c} r\\ \theta \end{array}],\;R=[\begin{array}{cc} \sigma_{r}^{2} & \sigma_{r\theta }^{2}\\ \sigma_{r\theta }^{2} & \sigma_{\theta }^{2} \end{array}]$$

where (*r,θ*) defines the distance and the direction of the observed landmark to the robot coordinate, and the observation covariance matrix is denoted as *R*.

If we get *i* (*i* > 1) observations at a time, the measured vector *Z* and its covariance matrix *R* can be described as:

(22) $$Z=[\begin{array}{c} z_{1}\\ z_{2}\\ \vdots \\ z_{i} \end{array}],\;R=[\begin{array}{cccc} R_{1} & 0 & 0 & 0\\ 0 & R_{2} & 0 & 0\\ 0 & 0 & \ddots & 0\\ 0 & 0 & 0 & R_{i} \end{array}]$$

Next, the transformation of the derived locations from the global Cartesian coordinate to the local robot side is as follows. Therefore, distinct map landmarks link with each other, and their relationships increase monotonically until their relative locations are known.

(23) $${\hat{z}}_{i}=h_{i}({\hat{X}}_{a}^{-})=[\begin{array}{c} \sqrt{{\left({\hat{x}}_{i}-{\hat{x}}_{v}\right)}^{2}+{\left({\hat{y}}_{i}-{\hat{y}}_{v}\right)}^{2}}\\ \arctan(\frac{{\hat{y}}_{i}-{\hat{y}}_{v}}{{\hat{x}}_{i}-{\hat{x}}_{v}})-{\hat{\varphi }}_{v} \end{array}]$$

If the measurement *z* associates with the predicted landmark position ${\left({\hat{x}}_{i},{\hat{y}}_{i}\right)}^{T}$ correctly, then we update the SLAM results.

(24) $${\hat{X}}_{a}^{+}={\hat{X}}_{a}^{-}+W_{i}v_{i}$$

(25) $$P_{a}^{+}=P_{a}^{-}-W_{i}S_{i}{W_{i}}^{T}$$

The measurements residual $v_{i}$, also called as innovation, which is the difference between the real perceived and estimated measurements, is defined as:

(26) $$v_{i}=z\;-\;h_{i}({\hat{X}}_{a}^{-})$$

with its covariance *S_i_*:

(27) $$S_{i}=HP_{a}^{-}H^{T}+R$$

and the Kalman gain *W_i_*:

(28) $$W_{i}=P_{a}^{-}H^{T}S_{i}^{-1}$$

where *H* represents the Jacobian matrix which linearizes the nonlinear measurements function h around the best estimation of the state ${\hat{X}}_{a}^{-}$. As for *H*, first _△_*x*, _△_*y*, *d*, *H_1_*, *H_2_*are defined in advance as follows:

(29) $$\Delta x={\hat{x}}_{i}-{\hat{x}}_{v},\;\Delta y={\hat{y}}_{i}-{\hat{y}}_{v}$$

(30) $$d=\sqrt{{\left({\hat{x}}_{i}-{\hat{x}}_{v}\right)}^{2}+{\left({\hat{y}}_{i}-{\hat{y}}_{v}\right)}^{2}}$$

(31) $$H_{1}={\frac{\partial h}{\partial X_{v}}|}_{{\hat{X}}_{a}^{-}}=\left[\begin{array}{ccc} -\frac{\Delta x}{d} & -\frac{\Delta y}{d} & 0\\ \frac{\Delta y}{d^{2}} & -\frac{\Delta x}{d^{2}} & -1 \end{array}\right],\;H_{2}={\frac{\partial h}{\partial X_{m}}|}_{{\hat{X}}_{a}^{-}}=\left[\begin{array}{cc} \frac{\Delta x}{d} & \frac{\Delta y}{d}\\ -\frac{\Delta y}{d^{2}} & \frac{\Delta x}{d^{2}} \end{array}\right]$$

(32) $$\begin{array}{ll} ∴H & ={\frac{\partial h}{\partial X_{a}}|}_{{\hat{X}}_{a}^{-}}=[\begin{array}{cccc} H_{1} & 0_{1} & H_{2} & 0_{2} \end{array}]\\ & =[\begin{array}{ccccccccccc} -\frac{\Delta x}{d} & -\frac{\Delta y}{d} & 0 & 0 & \cdots & 0 & \frac{\Delta x}{d} & \frac{\Delta y}{d} & 0 & \cdots & 0\\ \frac{\Delta y}{d^{2}} & -\frac{\Delta x}{d^{2}} & -1 & 0 & \cdots & 0 & -\frac{\Delta y}{d^{2}} & \frac{\Delta x}{d^{2}} & 0 & \cdots & 0 \end{array}] \end{array}$$

#### 4.6.4. Augmentation Stage

As the environment is explored, newly observed features need to be included in the generated map. Thus, we come up with an adequate solution for initializing new features. First and foremost, the system state and its covariance are extended by the new measurement $z_{new}$ and its covariance $R_{new}$, which are perceived relative to the robot coordinate:

(33) $${\hat{X}}_{aug}=[\begin{array}{c} {\hat{X}}_{a}\\ z_{new} \end{array}],\;P_{aug}=[\begin{array}{ccc} P_{v} & P_{vm} & 0\\ P_{vm}^{T} & P_{m} & 0\\ 0 & 0 & R_{new} \end{array}]$$

Here a transformation function $g_{i}$ is employed to change the polar perception $z_{new}$ into the global Cartesian coordinate. It consists of the present robot state ${\hat{X}}_{v}$ and the new sensor measurement $z_{new}$:

(34) $$g_{i}({\hat{X}}_{v},Z_{new})=[\begin{array}{c} x_{i}\\ y_{i} \end{array}]=[\begin{array}{c} x_{v}+r\cos(\theta +{\hat{\varphi }}_{v})\\ y_{v}+r\sin(\theta +{\hat{\varphi }}_{v}) \end{array}]$$

With the help of a linearized transformation $f_{i}$ function, the system augmented state is initialized to the correct values. The conversion formula $f_{i}$ is denoted as follows:

(35) $${\hat{X}}_{a}^{+}=f_{i}({\hat{X}}_{aug})=[\begin{array}{c} {\hat{X}}_{a}\\ g_{i}({\hat{X}}_{v},z_{new}) \end{array}]$$

(36) $$P_{a}^{+}=\nabla f_{x_{aug}}P_{aug}\nabla f_{x_{aug}}^{T}$$

where the sparse Jacobian matrix $\nabla f_{x_{aug}}$ is given by:

(37) $$\nabla f_{x_{aug}}={\frac{\partial f_{i}}{\partial X_{aug}}|}_{{\hat{X}}_{aug}}=[\begin{array}{ccc} I_{v} & 0 & 0\\ 0 & I_{m} & 0\\ G_{X_{v}} & 0 & G_{z_{new}} \end{array}]$$

and the Jacobian matrices $G_{X_{v}}$ and $G_{Z_{new}}$ are:

(38) $$G_{X_{v}}={\frac{\partial g_{i}}{\partial X_{v}}|}_{\left({\hat{X}}_{v},z_{new}\right)}=[\begin{array}{ccc} 1 & 0 & -r\sin(\theta +{\hat{\varphi }}_{v})\\ 0 & 1 & r\cos(\theta +{\hat{\varphi }}_{v}) \end{array}],\;G_{Z_{new}}={\frac{\partial g_{i}}{\partial Z_{new}}|}_{\left({\hat{X}}_{v},z_{new}\right)}\;=[\begin{array}{cc} \cos(\theta +{\hat{\varphi }}_{v}) & -r\sin(\theta +{\hat{\varphi }}_{v})\\ \sin(\theta +{\hat{\varphi }}_{v}) & r\cos(\theta +{\hat{\varphi }}_{v}) \end{array}]$$

The matrix multiplication of $P_{a}^{+}$ requires $O(n^{3})$ computation complexity where *n* is the number of landmarks on the map. Due to the sparseness of the Jacobian matrix, a much more efficient transformation can be implemented; it also only affects the block diagonal matrix of the newly observed landmark and off diagonal cross-correlations to the rest of the map.

(39) $$∴P_{a}^{+}=[\begin{array}{ccc} P_{v} & P_{vm} & P_{v}G_{X_{v}}^{T}\\ P_{vm}^{T} & P_{m} & P_{vm}^{T}G_{X_{v}}^{T}\\ G_{X_{v}}P_{v} & G_{X_{v}}P_{vm} & G_{X_{v}}P_{v}G_{X_{v}}^{T}+G_{z_{new}}R_{new}G_{z_{new}}^{T} \end{array}]$$

## 5. AEKF-SLAM and FastSLAM 2.0 Based Underwater Robotic Navigation Simulations

The following two MATLAB simulation experiments are carried out for both AEKF based SLAM and FastSLAM 2.0 for dense loop mapping and line mapping, which are executed by a generic autonomous robot measuring the environmental landmark points with a range-bearing sensor in a 2D area. Here, we assume that an AUV is observing fixed objects in an inertial reference system (INS) in order to enhance the localization performance, since the INS of an AUV suffers from drift. We can change the value of various parameters depending on the practical velocity of the Autonomous Underwater Vehicle (AUV), and the maximum perception range of the chosen sonars.

### 5.1. Dense Loop Map

**Figure 13:**
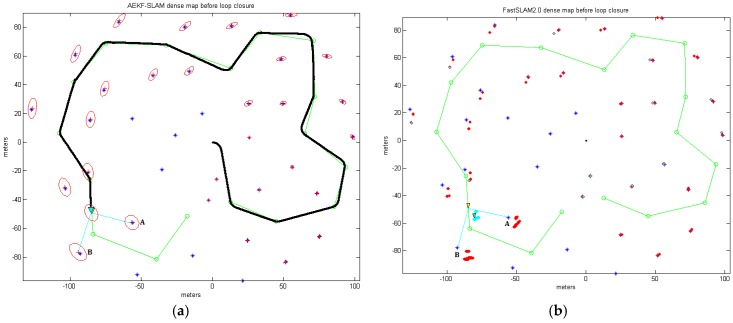
(**a**) The robot is observing the landmarks A and B in the AEKF-SLAM dense loop map; (**b**) The robot is getting measurements A and B in the FastSLAM 2.0 dense loop map.

The AEKF-SLAM and FastSLAM 2.0 simulation environment for the dense loop map is established as a 200 m × 200 m wide area with coordinates ranging from −100 m to 100 m. Indeed, 17 robot waypoints are arranged in form of a circle and 36 landmarks are randomly distributed near the robot trajectory as illustrated in Figure 13. We set the robot speed to 3 m/s, its deviation is 0.3 m/s, and its heading angle error is 3π/180 rad. The range and bearing of the robotic observation variance is 0.1 m/s and π/180 rad. The robot observes the surrounding features every 0.1 s with a sampling time of 0.0125 s. This leads to one image each 0.3 m with a maximum perception range of 30 m. As for the FastSLAM 2.0, there are 100 particles to estimate the 36 landmark positions and the robot pose, and we set 75 particles as the minimum number of effective particles before resampling.

Figure 13 depicts the 2D dense loop feature map built by our presented AEKF-SLAM algorithm and the conventional FastSLAM 2.0 approach, respectively, where the landmarks are arbitrarily distributed. The sensor scans for the landmark point positions are clearly to be seen, and few sightings are discarded by the statistical outlier rejection techniques [50], because they are out of the robotic perception distance. The rest denote distinct landmarks and are included into the stored map. The actual landmarks are depicted as blue stars (‘*’); green circles (‘∘’) are the robot waypoints, which are used for computing the robot steering angle. The landmark locations estimated by our AEKF-SLAM algorithm are drawn as the red crosses (‘+’) in Figure 13a, and the ellipse around every red cross illustrates the uncertainty covariance for the estimated landmark positions. The predicted robot trajectory is shown as the solid black line, leaded by a cyan triangle. Around this cyan triangle, there is a red ellipse, which denotes the covariance of the posterior AEKF estimate projected into the robot state. The larger it is, the more uncertain about the current robot pose is.

In Figure 13b, to clearly display the particles in the FastSLAM 2.0 dense loop experiment, they are drawn as the red dots (‘⋅’). The small cyan ellipses represent the covariances of max weight particle for estimating the robot pose and landmark positions. The true positions of the landmarks A and B, in Figure 13 dense loop maps, are at (−55.89 m, −55.92 m) and (−92.96 m, −77.73 m).

As the coordinate of the first detected landmark point is known with high precision, the uncertainty in the predicted robot state will reduce dramatically when the robot achieves the loop navigation and revisits this landmark. This situation results in the uncertainties of previously observed landmarks are also decreased. It is visible that our proposed AEKF is much more precise and more efficient than the FastSLAM 2.0 for dense loop mapping, since the AEKF estimates both the landmark positions and the robot pose faster and more accurately than the classic FastSLAM 2.0. Table 4 illustrates the comparisons of the processing time and the estimated landmark A and B positions in the dense loop map generated by our proposed AEKF-SLAM and FastSLAM 2.0.

Therefore, in the dense loop map, the standard deviations between the landmark positions A and B predicted by the AEKF-SLAM and the practical ones are:

$$\sigma_{A}=0.723,\;\sigma_{B}=1.921$$

The standard deviations between the landmark positions A and B approximated by the FastSLAM 2.0 and the real ones are:

$$\sigma_{A}=7.522,\;\sigma_{B}=10.021,$$

which are both 10 times larger than those of our proposed AEKF-SLAM. Besides, the AEKF-SLAM consumes less than one third processing time used by the conventional FastSLAM 2.0 for dense loop mapping. In conclusion, the AEKF-SLAM method has much better performances of localization and mapping accuracy with relatively low computational load.

### 5.2. Line Map

**Figure 14:**
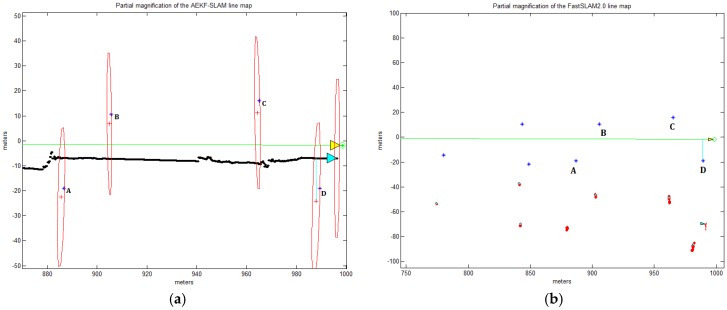
(**a**) Partial magnification of the AEKF-SLAM line map; (**b**) Partial magnification of the FastSLAM 2.0 line map.

The AEKF-SLAM and FastSLAM 2.0 simulation environment for the line map is an area of 1000 m × 800 m (from 0 m to 1000 m and from −400 m to 400 m). There are 27 landmarks and 4 robot waypoints. The velocity of the robot is 3 m/s, its variance is 0.3 m/s, the heading orientation error is 3π/180 rad. The range and bearing of the robotic measurement noise is 0.1 m/s and π/180 rad. As before, the sampling time is 0.0125 s, and the robot observes the surrounding features every 0.1 s, obtaining one observation every 0.3 m. The maximum perception distance is again 30 m. In Figure 14, the actual robot trajectory is along the *x*-axis (i.e., y=0), and the true landmark positions are indicated by blue stars (‘*’); four green circles (‘∘’) are the robot waypoints which are applied for measuring the robot steering angle. In Figure 14a, the line map which is built by the AEKF-SLAM, the red crosses (‘+’) are the estimated landmark locations, with red ellipses denoting their uncertainty covariance. The line map also depicts the estimated robot trajectory as the solid black line, guided by the cyan triangle. Around this cyan triangle, we can find a red ellipse, which represents the covariance of the posterior AEKF estimate projected into the robot state. The larger it is, the more uncertain is the current robot pose. As for the line map generated by the FastSLAM 2.0 in Figure 14b, we set 100 particles for estimating the 27 landmark positions and the robot pose, and 75 particles as the minimum number of effective particles before resampling. These particles are depicted as the red dots (‘⋅’) for a purpose of clear visualization. The small cyan ellipses represent the covariances of max weight particle for estimating the robot pose and landmark positions. In Figure 14, the true positions of the landmarks A, B, C and D are at (886.8 m, −19.04 m), (905.7 m, 10.68 m), (965.1 m, 16.08 m) and (989.4 m, −19.04 m).

In Figure 14a, it can be found that with the appearance of strange discontinuities in the estimated robot path, the inconsistency of the AEKF-SLAM algorithm becomes visibly evident. The jagged robot trajectory results from the inconsistency of variations in Jacobian linearization, given a large heading uncertainty. Over several successive observation updates, the estimated robotic pose shows dramatic jumps, which tend to be disproportionately large compared to the real measurement deviation (0.3 m/s). A related symptom also appears in the estimations of landmark positions. Again, the size of the landmark updates tends to be much larger than the actual observation noise (0.1 m/s). However, rather than exhibiting random motion in accord with the sensor noise, the average of landmark updates seems to be constrained to the line (x-axis). These symptoms manifest once the robot heading uncertainty becomes large, but they will not appear if the Jacobians are linearized about the true state. Note that these symptoms are not caused by numerical errors, since different numerical forms of the AEKF give identical results. These symptoms are simply due to a measurement trying to correct the robot heading, when the heading variance has been artificially reduced. Above all, it is evident that the AEKF-SLAM algorithm is much more reliable and efficient than the FastSLAM 2.0 for line mapping, since the AEKF-SLAM estimates both the landmark positions and the robot pose more precisely and faster than the classic FastSLAM 2.0. The comparisons of the computational complexity and the estimated landmark A, B, C and D positions in the simulated line map generated by the AEKF-SLAM and FastSLAM 2.0 are shown in Table 5.

As a consequence, in the line map, the standard deviations between the landmark positions A, B, C and D estimated by the AEKF-SLAM and the true ones are:

$$\sigma_{A}=3.724,\;\sigma_{B}=3.877,\;\sigma_{C}=4.983,\;\sigma_{D}=5.356$$

The standard deviations between the landmark positions A, B, C and D estimated by the FastSLAM 2.0 and the true ones are:

$$\sigma_{A}=54.099,\;\sigma_{B}=35.715,\;\sigma_{C}=32.697,\;\sigma_{D}=67.693,$$

which are all at least 6.5 times larger than those of the presented AEKF-SLAM. Even for the landmarks A and D, the standard deviations estimated by the FastSLAM 2.0 are both more than 12.5 times higher than for our method. Moreover, the computational time of the conventional FastSLAM 2.0 is nearly 6 times higher than that of the AEKF-SLAM for line mapping. As a conclusion, for both dense loop mapping and line mapping experiments, the AEKF-SLAM approach has the best performances of localization and mapping accuracy with relatively low computational cost.

### 5.3. SWARMs Seabed Mapping Use Case

Our presented AEKF-based underwater SLAM algorithm is going be tested and validated near Mangalia (Romania) in the Black Sea, around summer 2017 as a part of the European Smart and Networking Underwater Robots in Cooperation Meshes (SWARMs) project (http://www.swarms.eu/index.html). The SWARMs project aims at facilitating the creation, planning and execution of autonomous maritime and offshore operations by the use of surface and underwater vehicles, such as the Unmanned Surface Vehicles (USVs), Autonomous Underwater Vehicles (AUVs) and Remote Operating Vehicles (ROVs). The water temperature in Mangalia at a depth of 30 m would be 11.4 °C in July, the water salinity 18.2 g/L, and the sound speed would be 1475 m/s, and the sea bottom material is muddy gravel. All the underwater vehicles including the AUVs and ROVs that will be used in the SWARMs project are shown in the Table 6.

Localization and mapping is one of the key components that enables the autonomy of AUVs. Many sensor modalities can be used in this task, and the INS is one of the most common approaches. For precise maneuvering, an INS on board of the AUV calculates through dead reckoning the AUV position, acceleration, and velocity. Estimates can be made using data from an Inertial Measurement Unit (IMU). However, the INS suffers form a drift problem, which should be mitigated for a long-term operation of AUVs.

**Figure 15:**
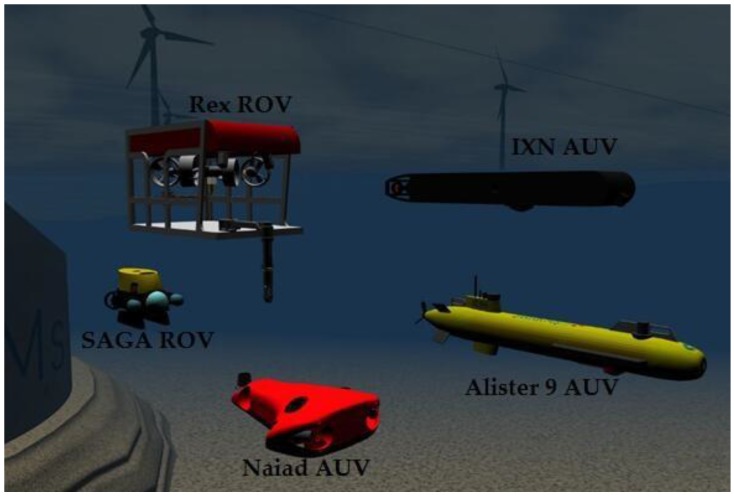
The simulated SWARMs vehicles.

In Figure 15, the following vehicles employed in the SWARMs project can be individually selected within the simulation environment and can be controlled by using a standard game controller: Alister 9 AUV provided by the ECA Group (Paris, France) [62], IXN AUV provided by the Ixion Industry & Aerospace SA company (Madrid, Spain) [63], Naiad AUV provided by Mälardalen University, SAGA ROV provided by Desistek Robotik Elektronik Yazilim company (Ankara, Turkey) [64], and an imaginary RexROV with a subsea manipulator, which is modeled similar to real existing ROVs. We have proved that it is possible and computationally feasible to concurrently simulate all the vehicles mentioned above in real time.

**Figure 16:**
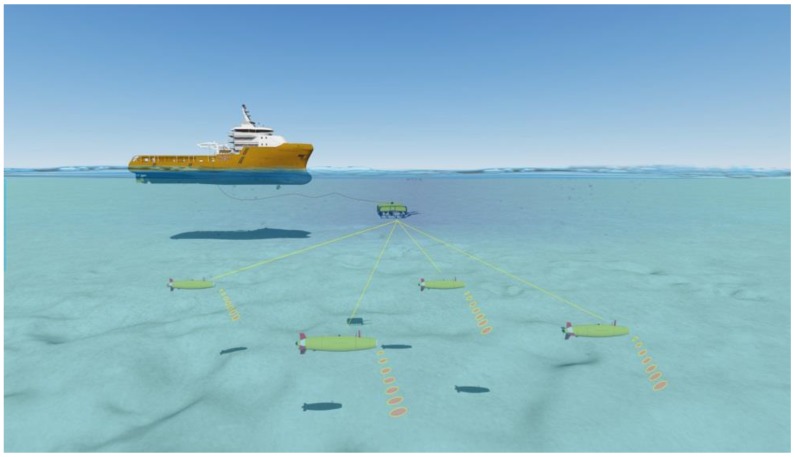
Link all the actors for landmark localization and seabed mapping in the SWARMs project.

There are several use cases in the SWARMs project, as e.g., use case No.5. (http://www.swarms.eu/usecases.html#case5), seabed mapping, which is composed of five stages. First, the USVs (Unmanned Surface Vehicles) pre-survey the area of interest. The USV survey path can be coordinated with the mother-ship or another USV to maximize the operational efficiency and the optimal multibeam overlap. Then, the AUVs are deployed over the seabed to be mapped. Next, the AUVs monitor and map the underwater environments with the assistance of their on-board navigation sensors to measure the distance from the AUVs to the seabed, they work cooperatively and follow the preplanned trajectories in order to cover the exploring region. Afterwards, the derived sonar data is transmitted from the AUVs to the ROV, which retransmits the perceived information via cable to the surface support vessel. Finally, real-time data mapping and monitoring will be accomplished. This procedure is illustrated in Figure 16. Here, the support vessel transports all the required equipments, robotic vehicles and supervises all the operations. The USVs perform a quick bathymetric pre-survey, and the bathymetry data collected from the USVs may thereafter be used to assist the planned track for the more detailed and high resolution mapping performed later by the AUVs. As for the AUVs, they are in charge of mapping the entire seabed as well as to characterize the subsea environment. The ROV plays a role as a relay between the support vessel and the AUVs to ease all underwater to surface communications. With a relatively short distance between the ROV and the AUVs, a faster real time seabed mapping and inspection is enabled. Real time maps could be presented to the operator computer. Operator intervention, such as lowering the speed of the AUVs, increases the resolution for detecting areas.

## 6. Conclusions and Future Work

### 6.1. Conclusions

In this work we focus on key topics related to SLAM applications in underwater environments. First of all, a detailed overview of currently used and popular solutions for the underwater SLAM problem has been given and characteristics like accuracy, robustness, computational complexity, etc. compared. Besides, different types of map representations have been compared regarding their suitability for a priori map localization, which are computational requirements, reliability, robustness, etc. In our case, the landmark map is chosen to represent the exploring underwater region to be explored. The robotic localization and map building process consists of generating the best estimation of the system states given the information available to the system. Thus, a new method, the AEKF-SLAM algorithm is provided. The main contribution of this work is to demonstrate this estimation theoretic solution to the underwater SLAM based navigation problem and to elucidate upon its mathematical structure. It is performed by storing the robot pose and the map landmarks in a single system state vector, and estimating the state parameters via a recursive, iterative, three-stage procedure comprising a prediction, an observation process (as in conventional EKF) and an augmentation state. The prediction phase deals with robot motion based on the incremental dead reckoning estimates, and it also increases the uncertainty of the robot pose estimate. The update stage occurs with the re-observation of already stored landmarks, improving the overall state estimate. When a landmark is detected for the first time, however, it is added to the state vector through an initialization process called state augmentation. The AEKF-SLAM based robotic underwater navigation algorithm emphasizes the identification of each sensor perception as a new landmark or a re-visited observed one. It can distinguish new landmarks from those already detected ones. With the assistance of the presented AEKF approach, the underwater robot achieves a more accurate and robust self-localization and mapping of the surrounding landmarks.

Compared with an efficient and classic approach, the FastSLAM 2.0, for both underwater dense loop mapping and line mapping simulation experiments, the provided AEKF-SLAM algorithm shows better performance in localization and mapping accuracy with relatively low computational cost. Moreover, the MATLAB simulation experiments performed for AEKF-SLAM based robotic dense loop mapping and line mapping behave much better in map management with respect to the landmark addition and removal to refrain from the long-term accumulation of errors and clutter in the generated map. By making those contributions available to the research community, we aim to facilitate the development, evaluation, comparison and consequently the improvement of the underwater SLAM algorithms.

### 6.2. Future Work

As a next step, within the framework of the European SWARMs project, our presented underwater AEKF-based SLAM algorithm will be tested and validated in underwater scenarios in the Black Sea with high currents speed (<1 m/s), low visibility (<1 m), high pressures (<15 atm), in a working area of about 1000–2000 m^2^. As for further enhancements of our present research, the future works include:

- Establishing a computationally tractable robotic underwater SLAM based navigation algorithm. The use of the full map covariance matrix at each stage in the underwater map generating process will lead to substantial computational problems. Hierarchical SLAM or sub-mapping methods build local maps of limited size, which bound the covariances and thus the linearization errors. Next, by incorporating the local maps into a global map or a hierarchy of global maps, the AEKF-SLAM based robotic navigation will be possible in large scenarios.
- Studying other state of the art approaches to the underwater SLAM problem for suitability, such as the graph-based SLAM and optimization-based SLAM. Afterwards, comparing their localization and mapping performances in terms of accuracy, robustness, computational complexity, etc. with those of the proposed AEKF-SLAM.
- Acquiring only one map of some part of the regions may not depict the global topology of the whole surveying area correctly, on account of the imaging geometry of the mapping devices. Integrating data measurements derived from the sonars and cameras to create a 3D subsea map, such as seafloor, exploring environment and artifacts.
- Simplifying and fusing two different resolution sonar maps by transform based algorithms. Employing the large scale medium resolution map to trigger detailed investigations of regions of interest in the local high resolution maps.
- Comparing the keypoint matching performances of the Scale Invariant Feature Transform (SIFT), the Affine Scale Invariant Feature Transform (ASIFT) and the Speed Up Robust Features (SURF), etc.

## Figures and Tables

**Table 1:** List of pros and cons of filtering approaches applied to the SLAM framework.

	KF/EKF	CEKF	IF	EM	PF
**Pros**	1. High convergence; 2. Handle uncertainty.	1. Reduced uncertainty; 2. Reduction of memory usage; 3. Handle large areas; 4. Increase map consistency.	1. Stable and simple; 2. Accurate; 3. Fast for high-D maps.	1. Optimal for map building; 2. Solve data association.	1. Handle nonlinearities; 2. Handle non-Gaussian noise.
**Cons**	1. Gaussian assumption; 2. Slow in high-D maps.	1. Need robust features; 2. Data acquisition; 3. Require multiple maps merging.	1. Data association; 2. Recover a state estimates; 3. Computationally expensive in high-D.	1. Inefficient, growing cost; 2. Unstable for large scenarios; 3. Only successful in map building.	1. Growth in complexity.

**Table 2:** The summary of state of the art underwater SLAM approaches.

Method [Reference]	Research Group	Underwater Vehicle	Sensor	Underwater Map	Filter
Daniel [22]	SRV ^1^	EcoMapper	Side Scan Sonar	Point Features	EKF
He [23]	SISE ^2^	C-Ranger	Forward Looking Sonar	Point Features	PF
Burguera [24]	SRV ^3^	Ictineu	Imaging Sonar	Vehicle Poses	EKF
Aulinas [25]	ViCoRob	SPARUS	Imaging Sonar	Point Features	EKF
Mallios [26]	ViCoRob	Ictineu	Imaging Sonar	Vehicle Poses	EKF
Barkby [27]	CAS	Sirus	Multibeam	Bathymetry	PF
Ribas [28]	ViCoRob ^4^	Ictineu	Imaging Sonar	Line Features	EKF
Fairfield [29]	WHOT	MBAUV	Sonar Beams	Evidence Grid	PF
Roman [30]	WHOI ^5^	JASON	Multibeam	Bathymetry	EKF
Fairfield [31]	CMU ^6^	DEPTHX	Sonar Beams	Evidence Grid	PF
Williams [32]	CAS ^7^	Oberon	Camera + Sonar	Point Features	EKF
Tena-Ruiz [33]	OSL ^8^	REMUS	Side Scan Sonar	Point Features	EKF
Williams [34]	ACFR ^9^	Oberon	Imaging Sonar	Point Features	EKF

^1^ SRV: Systems Robotics & Vision, Universitat de les Illes Balears, Spain; ^2^ SISE: School of Information Science and Engineering, Ocean University of China, China; ^3^ SPV: Systems, Robotics and Vision Group, Islas Baleares, Spain; ^4^ ViCoRoB: Computer Vision and Robotics group, Girona, Spain; ^5^ WHOI: Woods Hole Oceangraphic Institution, Woods Hole, MA, US; ^6^ CMU: Carnegie Mellon University, Pittsburgh, PA, US; ^7^ CAS: Centre of Excellence for Autonomous Systems, Sydney, Australia; ^8^ OSL: Ocean Systems Laboratory, Edinburgh, UK; ^9^ ACFR: Australian Center for Field Robotics, Sydney, Australia.

**Table 3:** The AEKF operations for achieving underwater SLAM.

Event	SLAM	AEKF
Robot Navigation	Robot Motion	AEKF Prediction
Sensor Detects Known Feature	Map Correction	AEKF Update
Sensor Detects New Feature	Landmark Initialization	State Augmentation
Map Corrupted Feature	Landmark Removal	State Reduction

**Table 4:** The comparisons of the computational time and estimated the landmark A, B positions in the dense loop map derived by the AEKF-SLAM and FastSLAM 2.0.

	Computational Time [s]	Estimated Landmark A [m]	Estimated Landmark B [m]
AEKF-SLAM	137.937051	(−56.61, −55.86)	(−94.46, −76.53)
FastSLAM 2.0	525.526820	(−51.38, −61.94)	(−87.06, −85.83)

**Table 5:** The comparisons of the computational time and estimated the landmark A, B, C, D positions in the line map derived by the AEKF-SLAM and FastSLAM 2.0.

	Computational Time [s]	Estimated Landmark A [m]	Estimated Landmark B [m]	Estimated Landmark C [m]	Estimated Landmark D [m]
AEKF-SLAM	99.837974	(885.5, −22.53)	(904.9, 6.886)	(964.4, 11.27)	(988, −24.21)
FastSLAM 2.0	594.113594	(879.4, −72.63)	(902.6, −46.26)	(961.7, −48.6)	(981.5, −86.27)

**Table 6:** The parameters of the underwater vehicles (AUVs and ROVs) employed in the SWARMs environmental sensing mission.

Platform	Circular Length (m)	Circular Width (m)	Circular Height (m)	Weight on Air (kg)
Alister 9 AUV	2.0	0.22	0.22	70
IXN AUV	1.9	0.5	0.3	150
Naiad AUV	0.84	0.6	0.25	30
SAGA ROV	0.42	0.33	0.27	10

## Abbreviations

The following abbreviations are used in this manuscript:

SLAM	Simultaneous Localization and Mapping
AEKF	Augmented Extended Kalman Filter
EKF	Extended Kalman Filter
AUVs	Autonomous Underwater Vehicles
CML	Concurrent Mapping and Localization
SONAR	SOund Navigation And Ranging
SSSs	Side-Scan Sonars
FLSs	Forward-Looking Sonars
IMU	Inertial Measurement Unit
DVL	Doppler Velocity Log
KF	Kalman Filter
PF	Particle Filter
EM	Expectation Maximization
CEKF	Compressed Extended Kalman Filter
IF	Information Filter
UKF	Unscented Kalman Filter
USAR	Urban Search and Rescue
GPS	Global Positioning System
LBL	Long Base Line
SBL	Short Base Line
USBL	Ultra Short Base Line
RSSI	Received Signal Strength Indication
RBPF	Rao-Blackwellised Particle Filtering
USVs	Unmanned Surface Vehicles
ROVs	Remote Operating Vehicles
SIFT	Scale Invariant Feature Transform
ASIFT	Affine Scale Invariant Feature Transform
SURF	Speed Up Robust Features
